# Clinical outcomes in individuals hospitalized with SARS-CoV-2 Delta variant (B.1.617.2) who had been vaccinated with Covishield (ChAdOx1) and Covaxin (BBV-152)

**DOI:** 10.1016/j.ijregi.2022.08.016

**Published:** 2022-09-05

**Authors:** Apoorva Munigela, Divya Tej Sowpati, Sasikala M, Sofia Banu, Archana Bharadwaj Siva, Jagadeesh Kumar V, Chandrasekhar Nutalapati, Ravikanth Vishnubhotla, Anand Kulkarni, Payel Mukherjee, Lamuk Zaveri, G.V. Rao, Karthik Bharadwaj Tallapaka, D. Nageshwar Reddy, CCMB COVID-19 Team, AIG Hospitals COVID-19 Vaccine Study Team

**Affiliations:** a AIG Hospitals, Internal Medicine, Gachibowli, Hyderabad, Telangana, India; b CSIR – Centre for Cellular and Molecular Biology, Habsiguda, Hyderabad, Telangana, India; c Asian Healthcare Foundation, Gachibowli, Hyderabad, Telangana, India; d Academy of Scientific and Industrial Research, Ghaziabad, India

**Keywords:** SARS-CoV-2, COVID-19, Covishield, Covaxin

## Abstract

**Background:**

Covishield (ChAdOx) and Covaxin (BBV-152) are the mainstream vaccines against severe acute respiratory syndrome coronavirus-2 (SARS-CoV-2) used in India and a few other countries.

**Objective:**

To assess the clinical outcomes of patients hospitalized with coronavirus disease 2019 (COVID-19) who had been vaccinated with either Covishield or Covaxin.

**Methods:**

This prospective, single-centre, observational cohort study of 1160 patients hospitalized with COVID-19 was conducted between April and June 2021. Severity of disease at admission and during hospitalization, requirement for intensive care unit (ICU) admission and ventilatory support, inflammatory markers (C-reactive protein, ferritin, lactate dehydrogenase, D-dimer), neutralizing antibody levels and mortality were assessed in vaccinated and unvaccinated patients.

**Results:**

More than 90% of patients in this study harboured the Delta variant (Pango lineage B.1.617.2) of SARS-CoV-2. Severity of disease at admission and during hospitalization (3.44% vs 7.51%; *P*=0.0032) and requirement for ICU admission and ventilatory support (2.83% vs 5.86%; *P*=0.0154) were significantly lower in vaccinated patients compared with unvaccinated patients. Vaccinated patients also had significantly (*P*<0.0001) higher antibody levels and lower inflammatory marker levels compared with unvaccinated patients. A subset of vaccinated, deceased patients mounted minimal antibody response [‘non-responders’: 4.53 (standard deviation 1.40) AU/mL].

**Conclusion:**

These results demonstrate the effectiveness of Covishield and Covaxin against severe disease in patients hospitalized with COVID-19 with breakthrough infections caused by the Delta variant. Strategies targeting non-responders are desirable to minimize morbidity and mortality.

## Highlights

• The Delta variant of severe acute respiratory syndrome coronavirus-2 is the predominant variant causing breakthrough infections in India.
• Disease severity was significantly lower in vaccinated individuals.
• Mortality reduced by >50% in fully vaccinated (two doses) individuals.
• Vaccinated individuals had higher antibody levels and lower inflammatory markers.
• Vaccinated deceased individuals mounted a minimal antibody response.

## Introduction

New variants of severe acute respiratory syndrome coronavirus-2 (SARS-CoV-2) continue to emerge as the virus spreads among hosts. A few of these variants, termed ‘variants of concern’, exhibit increased transmissibility or capacity to evade host immunity and are therefore associated with large outbreaks of COVID-19 worldwide (Campbell et al., 2021; Harvey et al., 2021). The Delta variant of SARS-CoV-2 (lineage B.1.617.2), first identified in late 2020 in India, is one such highly transmissible variant and has been associated with the large second wave of COVID-19 in India (Dhar et al., 2021). This variant has now spread to most countries and has become the largest circulating viral strain in the world (O'Toole et al., 2021). Vaccination is the most effective public health intervention to curtail the spread of SARS-CoV-2. However, SARS-CoV-2 infections are reported worldwide post vaccination (breakthrough infections), albeit in smaller numbers (Bergwerk et al., 2021; Juthani et al., 2021; Keehner et al., 2021). As most of the vaccines in current use were derived from viral strains that circulated in the early stage of the pandemic, it is imperative to assess their efficacy constantly against emerging variants, and to make changes to the vaccine composition as and when necessary (Moore and Offit, 2021). To date, in-vitro studies have revealed decreased neutralizing efficacy of the Pfizer-BioNTech vaccine (BNT162b2) and the Oxford-AstraZeneca vaccine (ChAdOx1) against the Delta variant (Davis et al., 2021), and limited data exist on the effectiveness of these vaccines against clinical outcomes in patients hospitalized with this variant. Studies on the effectiveness of vaccines such as Covaxin (BBV-152) are also limited. It is important to assess these other vaccines as they are being used to vaccinate a significant proportion of the population in different parts of the world. As such, the current study assessed the clinical outcomes of patients hospitalized with COVID-19 who had been vaccinated with one or two doses of either Covishield (ChAdOx1, Serum Institute of India) or Covaxin (BBV-152, Bharat Biotech International Limited) in comparison with unvaccinated patients.

## Methods

### Patient recruitment

**Figure 1:**
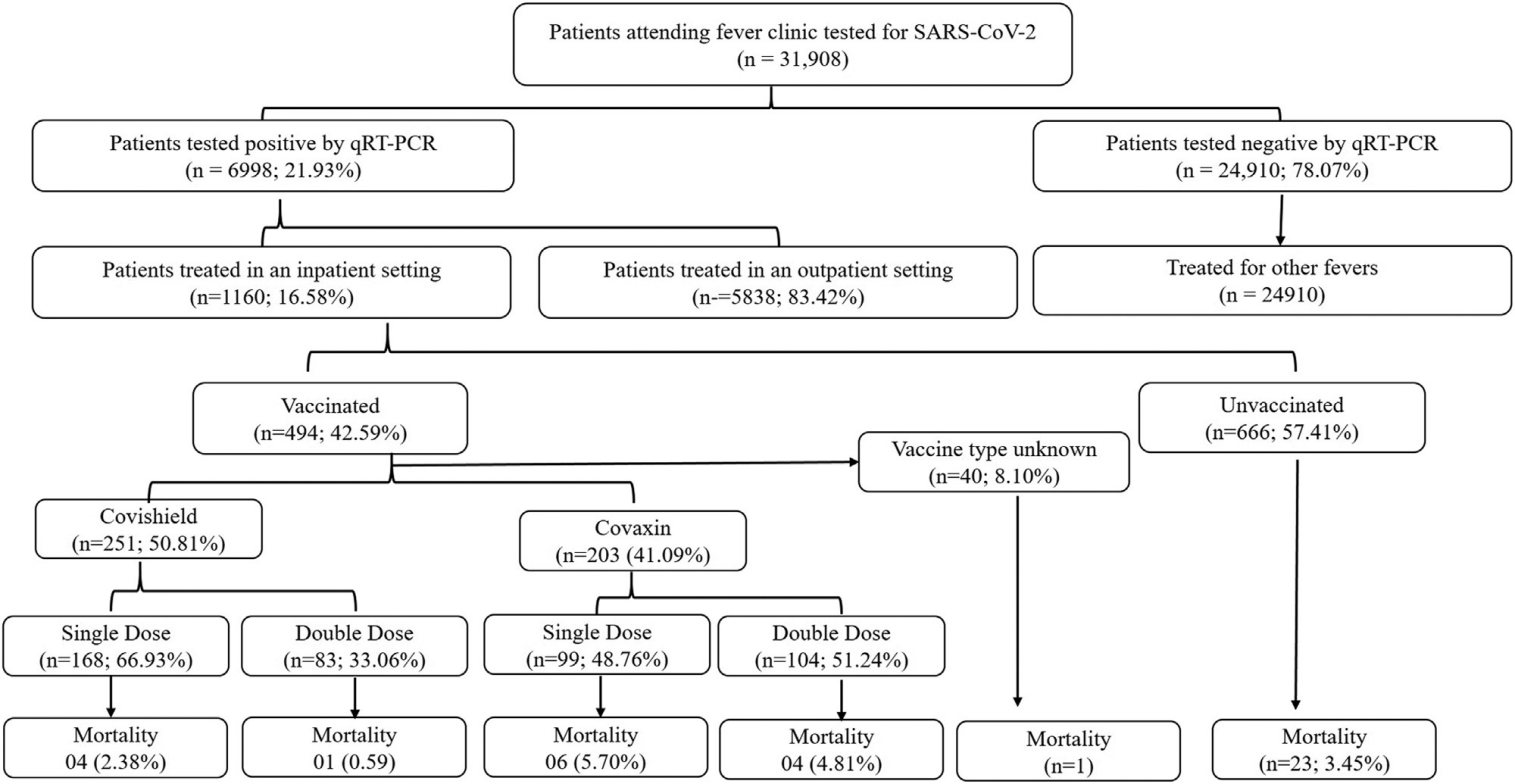
Consort diagram depicting the study design and enrolment of patients. SARS-CoV-2, severe acute respiratory syndrome coronavirus-2; qRT-PCR, quantitative reverse transcription polymerase chain reaction.

All patients with SARS-CoV-2 infection (*n*=1160) admitted to AIG Hospitals, a tertiary care referral hospital in Hyderabad, India, between April and June 2021 were enrolled in this study following approval from the institutional ethics committee (AIG/IEC-BH&R 12/02.2021-05) and informed consent (Figure 1). Patients with SARS-CoV-2 infection confirmed by reverse transcription polymerase chain reaction (RT-PCR), in-house or externally, were recommended for admission by the clinicians based on their clinical condition. Persistent fever for >72 h, extreme fatigue, low oxygen saturation (SpO_2_ <94%), risk of progressing to severe disease, and requirement for ventilatory support were the admission criteria. Unvaccinated patients with a history of natural infection were excluded from the study. Viral genome sequencing was performed in patients who tested positive for SARS-CoV-2 in the in-house laboratory. Clinical details such as symptom onset, duration of illness at the time of admission, and vaccination status were captured using a structured proforma. Details on vaccination date and vaccine type were obtained from patients, and were confirmed using the CoWIN app (developed by the Government of India for the vaccination process, https://www.cowin.gov.in) wherever possible. Patients who had received one or two doses of vaccine prior to symptom onset were considered ‘vaccinated’. Other patients were categorized as ‘unvaccinated’. Disease severity was assessed according to the current guidelines of the Indian Council of Medical Research. Patients with upper respiratory tract symptoms and without dyspnoea (or hypoxia), with or without fever, were considered to have mild disease. Patients with SpO_2_ of 90–93% (inclusive) were considered to have moderate disease, and patients with SpO_2_ <90% were considered to have severe disease (Clinical Guidance for Management of Adult COVID-19 Patients [icmr.gov.in]). Patients who required intensive care unit (ICU) admission at presentation (irrespective of oxygen saturation) were also included in this study; these patients were considered to have severe disease. All patients received standard treatment as per the guidelines of the Ministry of Health and Family Welfare, Government of India. Mortality occurring at any time during hospitalization was categorized as ‘in-hospital mortality’, and all patients were followed up for 28 days from admission to assess 28-day mortality.

### Laboratory work-up

Whole blood (5 mL in EDTA) was collected at admission from all patients for baseline laboratory parameters – complete haemogram, inflammatory markers [C-reactive protein (CRP), ferritin, lactate dehydrogenase (LDH), D-dimer] and neutralizing antibodies employing commercial kits following the manufacturers’ instructions. The complete haemogram was performed on a Beckman Coulter counter (Brea, CA, USA). The CRP level was evaluated by immunoassays on a VITROS 5600 (Ortho Diagnostics). The ferritin level was assessed by electrochemiluminescence (Roche Diagnostics, Mannheim, Germany), and the LDH level was measured using the pyruvate-lactate enzymatic method (Beckman Coulter AU500). The D-dimer level was estimated with an automated latex-enhanced immunoassay (Instrumentation Laboratories, Orangeburg, NY, USA). SARS-CoV-2 S1/S2 IgG neutralizing antibodies were enumerated on an automated Diasorin Liaison XL by chemiluminescence immunoassay. In this method, specific recombinant S1 and S2 antigens were coated on magnetic particles (solid phase), and mouse monoclonal antibodies to human immunoglobulin G (IgG) were linked to an isoluminol derivative. The antibodies in the serum bind to the solid phase and react with IgG to SARS-CoV-2. The luminescence generated is measured by a photomultiplier as relative light units, and the analyser calculates and provides the neutralizing antibody levels in arbitrary units (AU/mL). The detection limit is ≥3.8 AU/mL, and the samples with >15 AU/mL were considered positive for neutralizing antibodies. Sera with >400 AU/mL were single-fold diluted (1:10) prior to measurement (Bonelli et al., 2020).

### Whole-genome sequencing of SARS-CoV-2

Nasopharyngeal swabs were collected for viral RNA isolation and genome sequencing. RNA from patients with COVID-19 was sequenced using COVIDSeq (Illumina, San Diego, CA, USA) according to the manufacturer's protocol. Briefly, first-strand cDNA was synthesized using random hexamers and reverse transcriptase. Viral first-strand cDNA was amplified using two primer pools. After amplification, the two pools were combined, tagmented and purified. An amplification step was performed to add a 10-base index and sequencing adapter, and the samples were purified using paramagnetic beads to ensure the selection of fragments of the optimal size for sequencing. Purified samples were quantified, normalized and sequenced on a NovaSeq 6000 (Illumina) to obtain 100-bp paired-end reads.

Base calling was performed on raw image data using bcl2fastq v2.20.0.422 (Illumina). FastQC v0.11.9 (Babraham Bioinformatics, Cambridge, UK) was used to assess read quality. Trimmomatic was used to trim poor-quality bases and adapter sequences (Bolger et al., 2014). Mapping of reads to the indexed reference genome (NC_045512.2) was performed using HISAT2 v2.1.0 (Kim et al., 2019). Consensus sequences were generated using bcftools from the BAM file post alignment. Coverage across the genome was calculated using SAMtools depth. PANGO v3.0.5 was used to assign lineages to the consensus sequences. Mutations in the sequences were identified using Nextclade v1.1.0 (Hadfield et al., 2018).

### Definitions of comorbidities

Patients on antidiabetic medication, irrespective of the duration of therapy, were considered diabetic. Patients on antihypertensive medication were considered hypertensive. Patients with bronchial asthma, chronic obstructive pulmonary disease and interstitial lung disease were grouped together and considered to have pre-existing respiratory disease. Patients with a history of coronary interventions or cardiac rhythm disturbance interventions and pre-existing cerebrovascular events were grouped together and considered to have cardiovascular disease. Patients with clinically, biochemically or histologically proven liver disease were considered to have chronic liver disease. Patients who were on maintenance haemodialysis or who had an estimated glomerular filtration rate <60 for >3 months were considered to have chronic kidney disease. Patients who developed thrombotic complications, such as deep vein thrombosis, pulmonary thromboembolism, acute coronary syndromes, cerebrovascular accidents, transient ischaemic attack and embolism in the abdominal vessels, were considered to have thromboembolic complications. Patients with COVID-19 who required mechanical ventilation or non-invasive ventilation for respiratory failure were categorized as requiring ventilatory support. All patients received treatment as per standard protocols, and were followed up for their progress during hospitalization until discharge or death. All patients who recovered and were discharged were followed up for 28 days.

### Statistical analysis

Data were analysed using R software. Categorical data, including sex, comorbidities and outcomes of COVID-19, are expressed as *n* (%). Continuous variables, including age, biochemical variables (haemogram, liver function test and serum creatinine) and inflammatory variables (ferritin, LDH, CRP and D-dimer), are expressed as mean [standard deviation (SD)]. Categorical data were compared using Chi-squared test or Fisher's exact test, and continuous data were compared using Student's *t*-test. *P*<0.05 was considered to indicate significance. Data were visualized using the ggplot2 package in R.

## Results

### Demographics of patients hospitalized with COVID-19 with breakthrough infections

Of the 1160 patients admitted to hospital, 748 (64.48%) were male and 412 (35.52%) were female. Of these, 494 patients were vaccinated [mean age 58.52 (SD 13.08) years] and 666 patients were unvaccinated [mean age 47.49 (SD 14.96) years]. Vaccinated patients were significantly older than unvaccinated patients (*P*<0.001). Of the 494 vaccinated patients, 251 had received Covishield (*n*=168, one dose; *n*=83, two doses) and 203 had received Covaxin (*n*=99, one dose; *n*=104, two doses); for the remaining 40 patients, the vaccine type or number of doses was not available and these patients were excluded from further analysis. Of the 187 patients who had received two doses of vaccine (Covishield or Covaxin), 129 (68.98%) had received the second dose at least 2 weeks prior (i.e. they were ‘fully vaccinated’). Vaccinated patients had significantly more comorbidities such as diabetes, hypertension (*P*<0.0001) and cardiovascular disease (*P*<0.0001), than unvaccinated patients (Table 1).

**Table 1:** Clinical profiles, inflammatory markers and outcomes of vaccinated and unvaccinated patients with coronavirus disease 2019.

Parameter	Unvaccinated(*n*=666)	Vaccinated(*n*=494)	*P*-value
Age (years)	47.49 (SD 14.96)	58.52 (SD 13.08)	<2.2E-16
Females	235 (35.3%)95% CI 31–39	177 (35.8%)95% CI 31–40	0.8526
Diabetes/hypertension	243 (36.49%)95% CI 32–40	255 (51.62%)95% CI 47–56	3.25E-07
Hypothyroidism	66 (9.91%)95% CI 7–12	51 (10.32%)95% CI 7–13	0.844
Chronic kidney disease	15 (2.25%)95% CI 1–3	12 (2.43%)95% CI 1–4	0.8464
Chronic liver disease	9 (1.35%)95% CI – 0.6–2	5 (1.01%)95% CI 0.3–2	0.7872
Respiratory disease	14 (2.1%)95% CI 1–3	8 (1.62%)95% CI 7–3	0.6654
Cardiovascular disease	18 (2.7%)95% CI 1–4	37 (7.49%)95% CI 5–10	0.0002188
Malignancy	4 (0.6%)95% CI 0.1–1	4 (0.81%)95% CI 0.2–2	0.7294
C-reactive protein (mg/L)	45.74 (SD 55.63) (*n*=617)	45.63 (SD 56.52) (*n*=460)	0.9748
Ferritin (ng/mL)	561.54 (SD 706.07) (*n*=611)	392.57 (SD 448.83) (*n*=460)	2.09E-06
D-dimer (ng/mL)	436.04 (SD 739.59) (*n*=617)	438.7 (SD 845.74) (*n*=460)	0.957
Lactate dehydrogenase (U/L)	644.35 (SD 294.13) (*n*=539)	559.57 (SD 324.45) (*n*=401)	4.14E-05
Total leukocytes (cells/mm^3^)	7096.6 (SD 3587.72) (*n*=617)	7145.59 (SD 3536.78) (*n*=454)	0.8238
Neutrophils (cells/mm^3^)	5295.18 (SD 3364.82) (*n*=617)	5163.52 (SD 3223.31) (*n*=454)	0.5169
Lymphocytes (cells/mm^3^)	1334.91 (SD 673.93) (*n*=617)	1400.66 (SD 648.44) (*n*=453)	0.1074
Platelets (thousands/mm^3^)	229.74 (SD 83.18) (*n*=617)	220.74 (SD 74.62 (n=454)	0.06373
Baseline neutralizing antibodies (AU/mL)	51.47 (SD 297.42) (*n*=536)	544.56 (SD 1258.36) (*n*=428)	1.62E-14
Thrombotic complications	19 (2.85%)95% CI 1–4	11 (2.23%)95% CI 1–3	0.5775
Acute kidney injury	30 (4.5%)95% CI 3–6	24 (4.86%)95% CI 3–7	0.7799
Renal replacement therapy	5 (0.75%)95% CI 0.2–1	6 (1.21%)95% CI 0.4–2	0.5431
Ventilatory support	39 (5.86%)95% CI 4–7	14 (2.83%)95% CI 1–4	0.01545
Severe disease/ICU required at admission	50 (7.51%)95% CI 5–9	17 (3.44%)95% CI 2–5	0.003261
ICU required during hospital stay	42 (6.31%)95% CI 4–8	22 (4.45%)95% CI 2–6	0.1942
Death	23 (3.45%)95% CI 2–5	16 (3.23)95% CI 1–5One dose: 9 (3.24%)95% CI 0.8–3Two doses: 7 (3.24%)95% CI 0.5–2	111

ICU, intensive care unit; SD, standard deviation; CI, confidence interval.

### B.1.617.2 (Delta variant) was the predominant variant

**Figure 2:**
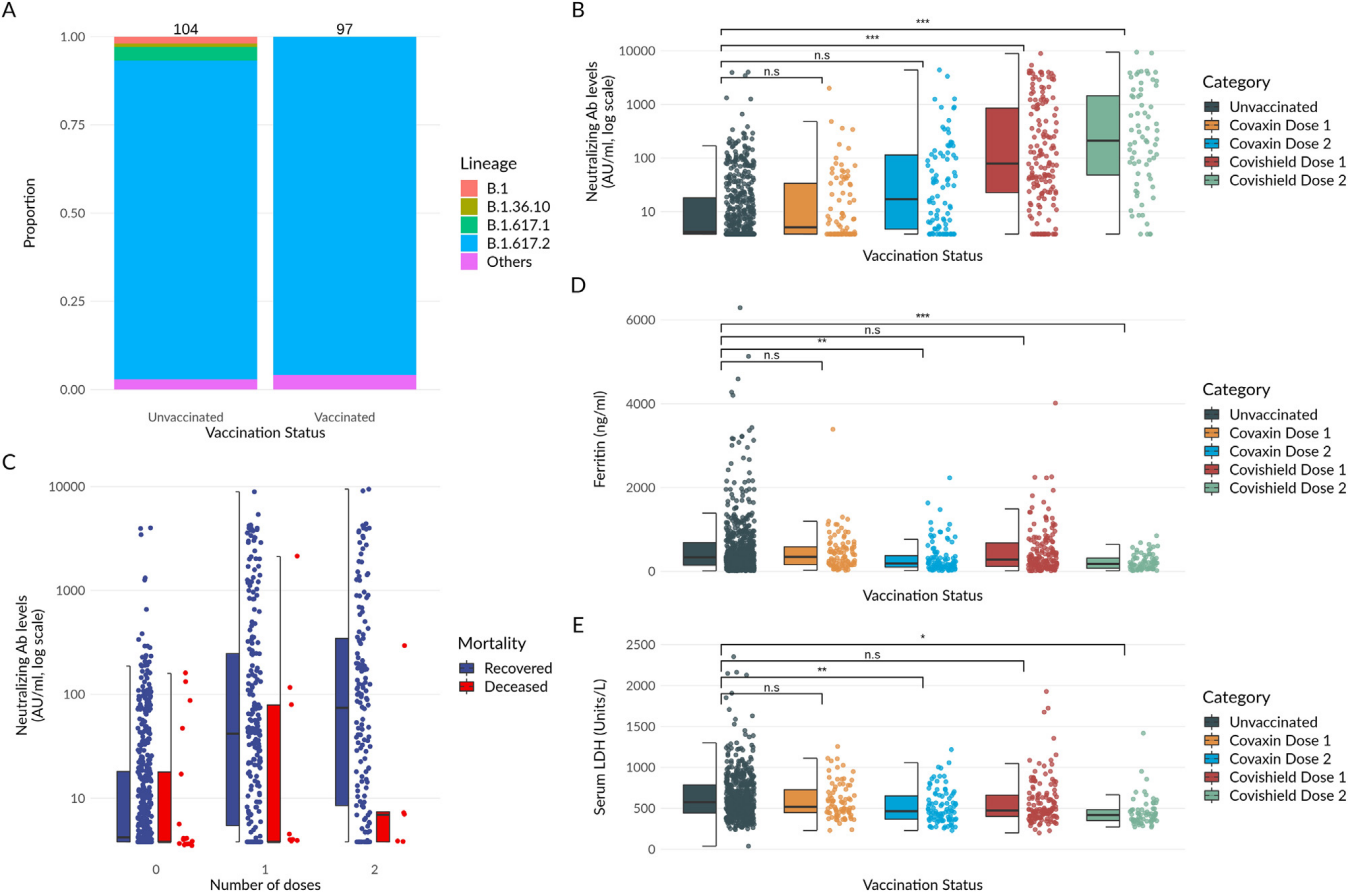
(A) A stacked bar plot representing the proportions of various severe acute respiratory syndrome coronavirus-2 (SARS-CoV-2) lineages in vaccinated and unvaccinated patients. The total number of genomes in a group is indicated on the top of the bar. (B) Neutralizing antibody (Ab) levels in unvaccinated and vaccinated patients with breakthrough infections (Covaxin and Covishield; one and two doses). (C) Neutralizing Ab levels in recovered and deceased patients. (D and E) Serum ferritin and lactate dehydrogenase (LDH) levels in vaccinated and unvaccinated patients with breakthrough infections. Significant differences are indicated with an asterisk.

The viral genome could be sequenced from the nasopharyngeal swabs obtained from 201 patients (vaccinated: *n*=97; unvaccinated: *n*=104) during the study period. More than 90% of patients in both groups were infected with the Delta variant (Pango lineage B.1.617.2): 95.87% (93/97) in the vaccinated group and 90.60% (94/104) in the unvaccinated group (Figure 2A). This was comparable to the prevalence of the Delta variant in the community during this period (Figure S1, see online supplementary material).

### S1/S2 spike IgG neutralizing antibodies in breakthrough infections

Vaccinated patients showed significantly higher levels of neutralizing antibodies compared with unvaccinated patients [544.56 (SD 1258.36) AU/mL vs 51.47 (SD 297.42) AU/mL; *P*<0.001; Figure 2B]. Among the unvaccinated patients, 33 of 666 (18 male and 15 female) patients mounted a significant antibody response (>100 AU/mL). However, they were found to have IgM antibodies [IgM 7.41 (SD 8.17), S/C cut-off index 1.0], suggesting that these were acute infections. Sex did not have any significant effect on the antibody levels measured (Figure S2, see online supplementary material). A significant difference (*P*=0.01) in the neutralizing antibody response was observed between fully vaccinated (two doses) patients who died [162.65 (SD 269.06) AU/mL] and those who recovered [606.82 (SD 1406.89) AU/mL; Figure 2C].

### Lower levels of inflammatory markers observed in vaccinated patients

Among the inflammatory markers, serum ferritin [392.57 (SD 448.83) ng/mL vs 561.54 (SD 706.07) ng/mL; *P*<0.001] and LDH [559.57 (SD 324.45) U/L vs 644.35 (SD 294.13) U/L; *P*<0.001] were lower in vaccinated patients compared with unvaccinated patients (Table 1, Figure 2D and 2E).

### Severity of disease was lower in vaccinated patients

Fewer vaccinated patients presented with severe disease (SpO_2_ <90%/ICU requirement) at admission compared with unvaccinated patients (3.44% vs 7.51%; *P*=0.0032) (Table 1). Furthermore, fewer vaccinated patients required ventilatory support (2.83% vs 5.86%; *P*=0.0154) (Table 1). The incidence rates of acute kidney injury, renal replacement therapy and thrombotic complications were similar between the groups (Table 1). Mortality was similar in patients vaccinated with either one (9/278=3.24%) or two (7/216=3.24%) doses of vaccine. However, in the subset of patients who had received their second dose at least 2 weeks prior, the mortality rate was approximately 50% lower (2/129=1.55%) than in unvaccinated patients (23/666=3.45%) (Table 1). No significant differences were found when patients were categorized based on vaccine type and dose (Table 2). In addition, only one vaccinated patient died after 28 days of admission. Thus, the 28-day mortality rates were also consistent with the above observations.

**Table 2:** Comparison of clinical characteristics between unvaccinated and vaccinated patients (categorized by one and two doses).

Parameter	Unvaccinated	One dose of Covaxin	*P*-value	One dose of Covishield	*P*-value	Two doses of Covaxin	*P*-value	Two doses of Covishield	*P*-value
Age (years)	47.49 (SD 14.96) (*n*=666)	56.93 (SD 14.03) (*n*=99)	5.00E–09	56.33 (SD 12.33) (*n*=168)	6.20E–12	58.12 (SD 12.02) (*n*=104)	1.20E–11	64.76 (SD 12.76) (*n*=83)	2.00E-16
C-reactive protein (mg/L)	45.74 (SD 55.63) (*n*=617)	46.15 (SD 46.87) (*n*=94)	1	51.84 (SD 66.10 (*n*=155)	1	45.51 (SD 60.4) (*n*=101)	1	33.46 (SD 37.12) (*n*=75)	0.66
Ferritin (ng/mL)	561.54 (SD 706.07) (*n*=611)	455.41 (SD 446.52) (*n*=93)	0.6018	478.91 (SD 553.97) (*n*=158)	0.6018	331.91 (SD 385.70) (*n*=99)	0.0051	223.11 (SD 187.13) (*n*=75)	7.20E-05
D-dimer (ng/mL)	436.04 (SD 739.59) (*n*=617)	491.57 (SD 1078.88) (*n*=93)	1	449.66 (SD 716.96) (*n*=155)	1	334.12 (SD 561.12) (*n*=101)	1	481.38 (SD 1129.33) (*n*=75)	1
Lactate dehydrogenase (U/L)	644.35 (SD 294.13) (*n*=539)	591.73 (SD 219.25) (*n*=81)	0.7927	588.09 (SD 343.65) (*n*=138)	0.4525	521.73 (SD 206.9) (*n*=90)	0.0051	516.14 (SD 506.71) (*n*=64)	0.0157
Total leukocytes (cells/mm^3^)	7096.6 (SD 3587.72) (*n*=617)	6335.87 (SD 3246.69) (*n*=92)	0.378	7468.46 (SD 3807.04) (*n*=149)	0.852	6624.75 (SD 2909.69) (*n*=101)	0.852	7896.15 (SD 3541.06) (*n*=78)	0.378
Neutrophils (cells/mm^3^)	5295.18 (SD 3364.82) (*n*=617)	4610.53 (SD 3031.32) (*n*=92)	0.57	5504.68 (SD 3486.9) (*n*=149)	1	4765.17 (SD 2738.71) (*n*=101)	0.8	5456.62 (SD 3250.23) (*n*=78)	1
Lymphocytes (cells/mm^3^)	1334.91 (SD 673.93) (*n*=617)	1272.08 (SD 654.87) (*n*=92)	1	1424.46 (SD 647.29) (*n*=149)	0.84	1356.85 (SD 564.33) (*n*=101)	1	1532.16 (SD 741.88) (*n*=77)	0.13
Platelets (thousands/mm^3^)	229.74 (SD 83.18) (*n*=617)	205.51 (SD 58.77) (*n*=92)	0.065	227.45 (SD 79.51) (*n*=149)	1	221.58 (SD 82.17) (*n*=101)	1	219.74 (SD 64.83) (*n*=78)	1
Baseline neutralizing antibodies (AU/mL)	51.47 (SD 297.4) (*n*=536)	55.57 (SD 222.81) (*n*=88)	0.9652	755.29 (SD 1374.89) (*n*=156)	< 2e–16	196.51 (SD 606.56) (*n*=92)	0.3495	1141.39 (SD 1910.8) (*n*=69)	<2e-16
Diabetes/hypertension	243 (36.48%)95% CI 32–40	54 (54.55%)95% CI 44–64	0.0059	58 (55.77%)95% CI 27–42	0.0023	86 (51.19%)95% CI 74–89	0.0045	47 (56.63%)95% CI 45–67	0.0045
Cardiovascular disease	18 (2.7%)95% CI 1–4	6 (6.06%)95% CI 2–12	0.779	7 (6.73%)95% CI 1–8	0.522	14 (8.33%)95% CI 7–21	0.023	7 (8.43%)95% CI 3–16	0.131
Respiratory disease	14 (2.1%)95% CI 1–3	0 (0%)95% CI 0–3	1	2 (1.92%)95% CI 1–4	1	3 (1.79%)95% CI 6–8	1	3 (3.61%)95% CI 7–10	1
Malignancy	4 (0.6%)95% CI 1–1	2 (2.02%)95% CI 0.2–7	1	1 (0.6%)95% CI 0.01–3	1	0 (0%)95% CI 0–3	1	1 (1.2%)95% CI 0.03–6	1
Kidney disease	15 (2.25%)95% CI 1–3	1 (1.01%)95% CI 3–5	1	5 (2.98%)95% CI – 0.9–6	1	2 (1.92%)95% CI 2–6	1	4 (4.82%)95% CI 1–11	1
Chronic liver disease	9 (1.35%)95% CI 0.6–2	0 (0%)95% CI 0–3	1	4 (2.38%)95% CI 0.6–5	1	0 (0%)95% CI 0–3	1	1 (1.2%)95% CI 0.03–6	1
ICU required at admission	28 (4.2%)95% CI 2–6	1 (1.01%)95% CI 0.03–5	1	2 (1.19%)95% CI 0.1–4	0.62	3 (2.88%)95% CI 0.6–8	1	0 (0%)95% CI 0–3	0.62
ICU required during hospitalization	42 (6.31%)95% CI 4–8	6 (6.06%)95% CI 2–12	1	7 (4.17%)95% CI 1–8	1	6 (5.77%)95% CI 2–12	1	3 (3.61%)95% CI 0.7–10	1
Ventilatory support	39 (5.86%)95% CI 4–7	3 (3.03%)95% CI 0.6–8	1	3 (1.79%)95% CI 0.3–5	0.29	6 (5.77%)95% CI 2–12	1	2 (2.41%)95% CI 0.2–8	1
Thrombotic complications	19 (2.85%)95% CI 1–4	3 (3.03%)95% CI 0.6–8	1	2 (1.19%)95% CI 0.1–4	1	3 (2.88%)95% CI 0.6–8	1	3 (3.61%)95% CI – 0.7–10	1
Acute kidney injury	30 (4.5%)95% CI 3–6	4 (4.04%)95% CI 1–10	1	10 (5.95%)95% CI 2–10	1	4 (3.85%)95% CI 1–9	1	4 (4.82%)95% CI 1–11	1
Renal replacement therapy	5 (0.75%)95% CI 0.2–1	0 (0%)95% CI 0–0.3	1	3 (1.79%)95% CI 0.3–5	1	2 (1.92%)95% CI 0.2–6	1	1 (1.2%)95% CI 0.03–6	1
Death	23 (3.45%)95% CI 2– 5	5 (5.05%)95% CI 1–11	1	4 (2.38%)95% CI 0.6–5	1	5 (4.81%)95% CI 1–10	1	1 (1.2%)95% CI 0.03–6	1

ICU, intensive care unit; SD, standard deviation; CI, confidence interval.

### Poor antibody response in deceased vaccinated patients

Many (10/15; 8/10 Covaxin recipients and 2/5 Covishield recipients) of the deceased vaccinated patients had no antibody response [‘non-responders’: 4.53 (SD 1.40) AU/mL]. A higher mortality rate was observed in vaccinated patients with no antibody response (7.77% vs 1.27% in Covaxin recipients; 4.76% vs 1.61% in Covishield recipients) compared with those who tested positive for neutralizing antibodies.

### Comparison of immune responses, inflammatory markers and mortality between Covaxin and Covishield

The levels of S1/S2 spike IgG neutralizing antibodies were significantly (*P*<0.0001) higher in hospitalized patients who had received Covishield [one dose 755.29 (SD 1374.89) AU/mL, two doses 1141.39 (SD 1910.80) AU/mL] compared with unvaccinated patients [51.47 (SD 297.42) AU/mL]. No significant (*P*=0.965) difference in the levels of S1/S2 spike IgG neutralizing antibodies was observed after the first dose of Covaxin [55.57 (SD 222.81) AU/mL]. However, a trend towards a higher level of S1/S2 spike IgG neutralizing antibodies was observed after the second dose of Covaxin [196.51 (SD 606.56) AU/mL), although this was not significant (*P*=0.349). There was a significant difference (*P*=0.0001) in antibody levels between Covishield [one dose 755.29 (SD 1374.89) AU/mL, *n*=156; two doses 1141.39 (SD 1910.80) AU/mL, *n*=69] and Covaxin [one dose 55.57 (SD 222.81) AU/mL, *n*=88; two doses 196.51 (SD 606.56) AU/mL, *n*=92). Higher levels of antibodies were noted in the Covishield group compared with the Covaxin group for both one and two doses.

Among the inflammatory markers, serum ferritin and LDH levels were significantly lower after the second dose of both vaccines (Figure 2D and 2E), while CRP [unvaccinated: 45.74 (SD 55.63) mg/L; Covaxin: 45.51 (SD 60.40) mg/L; Covishield: 33.46 (SD 37.12) mg/L] and D-dimer levels [unvaccinated: 436.04 (SD 739.59) ng/mL; Covaxin: 334.12 (SD 561.12) ng/mL; Covishield: 481.38 (SD 1129.33) ng/mL] did not change significantly, even after the second dose of vaccine. When patients who had received two doses of either of the vaccines were compared, those who received two doses of Covishield had significantly lower levels of serum ferritin (*P*=0.01534) and significantly higher total leukocyte counts (*P*=0.0111) and levels of neutralizing antibodies (*P*=0.0001) (Table S1, see online supplementary material; Table 2).

Of the 15 deceased patients with breakthrough infections where the vaccine type was known, five (33.33%) had received Covishield and 10 (66.67%) had received Covaxin. Of the five patients who had received Covishield, one patient had received two doses and four patients had received one dose, while of the 10 patients who had received Covaxin, four patients had received two doses and six patients had received one dose. The difference in mortality rates between the groups did not reach significance (Table 2).

## Discussion

In this cohort study, the primary aim was to assess the effectiveness of Covishield and Covaxin in evoking a neutralizing antibody response and affecting the clinical outcomes in hospitalized patients with breakthrough infections harbouring the Delta variant (B.1.617.2). Vaccinated patients were significantly older, as individuals aged >60 years and >45 years with comorbidities were prioritized in the vaccination drive in accordance with the guidelines of the Government of India. Vaccinated patients had significantly more comorbidities than unvaccinated patients. The Delta variant was the predominant variant in both vaccinated and unvaccinated patients, and rates were comparable to the prevalence of the Delta variant in the community during this period. Hence, it may be safe to assume that the proportions hold true in the remaining samples in which viral genome sequencing could not be performed.

This study found reduced disease severity in vaccinated patients compared with unvaccinated patients. This corroborates the recent results demonstrating reduced disease severity and hospitalization in breakthrough infections (Bahl et al., 2021; Brosh-Nissimov et al., 2021; Gupta et al., 2021). In addition, the present results also support the findings of studies that evaluated the association of mRNA vaccines [mRNA-1273 (Moderna) and BNT162b2 (Pfizer-BioNTech)] with reduced hospitalization, severe disease and mortality (Griffin et al., 2021; Tenforde et al., 2021). A higher mortality rate was expected in older patients and those with comorbidities; however, no significant difference was found in comparison with the unvaccinated group and the younger age group, pointing to the benefits of vaccination in the former group. Within the vaccinated group, a trend towards higher antibody levels and lower inflammatory marker levels was noted in patients who had received two doses of vaccine compared with those who had received one dose (Figure 2B–D; Figure S3, see online supplementary material). Higher neutralizing antibody response and lower inflammatory markers suggest early neutralization of the virus, thereby preventing an aberrant inflammatory response. This result is in contrast to several earlier studies in patients hospitalized with COVID-19, which reported a positive correlation between disease severity and antibody levels (Chen et al., 2020; Hu et al., 2020; Yu et al., 2021). Higher antibody levels in vaccinated patients are, therefore, likely to be a good prognostic factor, in contrast to unvaccinated patients. The present study found minimal antibody response at admission in a few vaccinated, deceased patients. Recent studies have suggested that the dynamics of the antibody response may be more important than the actual antibody levels, with severe outcomes being noted in individuals with a delayed response (Rydyznski Moderbacher et al., 2020; Lucas et al., 2021). Therefore, low antibody levels in vaccinated patients may be considered a poor prognostic marker, warranting confirmation in larger cohorts. Alternate strategies targeting these ‘non-responders to vaccination’ also need to be explored further.

The neutralizing antibody response and total leukocyte count were significantly higher, and the serum ferritin level was significantly lower in patients who had received Covishield (two doses) than in those who had received Covaxin (two doses) (Table S1 and Figure S4, see online supplementary material; Table 2). However, the differences in severity/mortality between the groups was not significant. This suggests a likely role for other pathways in the immune system, apart from neutralizing antibody responses, in determining the efficacy of vaccine-induced immunity. A larger cohort may bring further clarity to these observations.

This study has a few limitations. SARS-CoV-2-positive patients were admitted based on in-house RT-PCR results or reports generated in other laboratories. Therefore, viral sequencing could only be performed on 201 nasopharyngeal swabs collected in-house. Although vaccination information was confirmed on the CoWIN app wherever possible, data from a few patients were not complete and hence could not be included in the analyses. Several laboratory parameters had a wide range of distribution. Neutralizing antibody levels were not measured serially in the patients. Finally, as this was a hospital-based study, there was inherent bias towards inclusion of high risk/severely affected patients.

In summary, the Delta variant of SARS-CoV-2 was the predominant cause of breakthrough infections during the second wave of the pandemic in India. The results from this study indicate that full vaccination (two doses) offers protection against the Delta variant, resulting in a reduction in disease severity among hospitalized patients. Furthermore, assessment of the antibody response in hospitalized patients can be used to define prognosis, and identification of ‘non-responders to vaccination’ may be an important step to minimize breakthrough infections and associated mortality.

## CCMB COVID-19 Team

Sreenivas Ara, Sumedha Avadhanula, Himasri Bollu, Sai Krishna Jandhyala, Onkar Kulkarni, Vidhyadhari Methuku, Sai Priya Nurkurthy, Valli Undamatla, Shreekant Verma, Amareshwar Vodapalli.

## AIG Hospitals COVID-19 Vaccine Study Team

Krishna Vemula, Jayashree Kanna, Kavitha Dharavath, Bratati Maity, Deepika Gujjarlapudi, Sadhana Yelamanchili.

## Data availability

Deidentified data from this study are deposited on GitHub, and are available at https://github.com/sowpati/ClinicalOutcomes_PostVaccination.

## Code availability

All R codes used for analysis and visualization are deposited on GitHub, and are available at https://github.com/sowpati/ClinicalOutcomes_PostVaccination under a GNU License.

## Conflict of interest statement

None declared.

## Appendix Supplementary materials

Image, application 1
