# Supplementary material for: Clinical outcomes in individuals hospitalized with SARS-CoV-2 Delta variant (B.1.617.2) who had been vaccinated with Covishield (ChAdOx1) and Covaxin (BBV-152)
Source: IJID Reg. 2022 Sep 5;5:104–10. doi: 10.1016/j.ijregi.2022.08.016 (PMC9444261; doi:10.1016/j.ijregi.2022.08.016)
Supplement: Supplementary file 1 [file mmc1.docx]

**Supplementary Information**

**Clinical outcomes in Covishield (ChAdOx1) and Covaxin (BBV-152) vaccinated individuals hospitalized with the Delta variant (B.1.617.2)**

Apoorva Munigela^1^, Divya Tej Sowpati^2^, Sasikala M^3^, Sofia Banu^2,4^, Archana Bharadwaj Siva^2^, Jagadeesh Kumar V^1^, Chandrasekhar Nutalapati^1^, Ravikanth Vishnubhotla^3^, Anand Kulkarni^1^, Payel Mukherjee^2^, Lamuk Zaveri^2^, CCMB COVID-19 Team^2^, AIG Hospitals COVID-19 Vaccine study Team^1^, GV Rao^1^, Karthik Bharadwaj Tallapaka^2,^*, D Nageshwar Reddy^1,^*

1 AIG Hospitals, Internal Medicine, Mindspace Rd, Gachibowli, Hyderabad, Telangana 500032

2 CSIR - Centre for Cellular and Molecular Biology, Uppal Road, Habsiguda, Hyderabad 500007

3 Asian Healthcare Foundation, Mindspace Rd, Gachibowli, Hyderabad, Telangana 500032

4 Academy of Scientific and Industrial Research (AcSIR), Ghaziabad - 201002, India

Apoorva Munigela and Divya Tej Sowpati contributed equally to this work


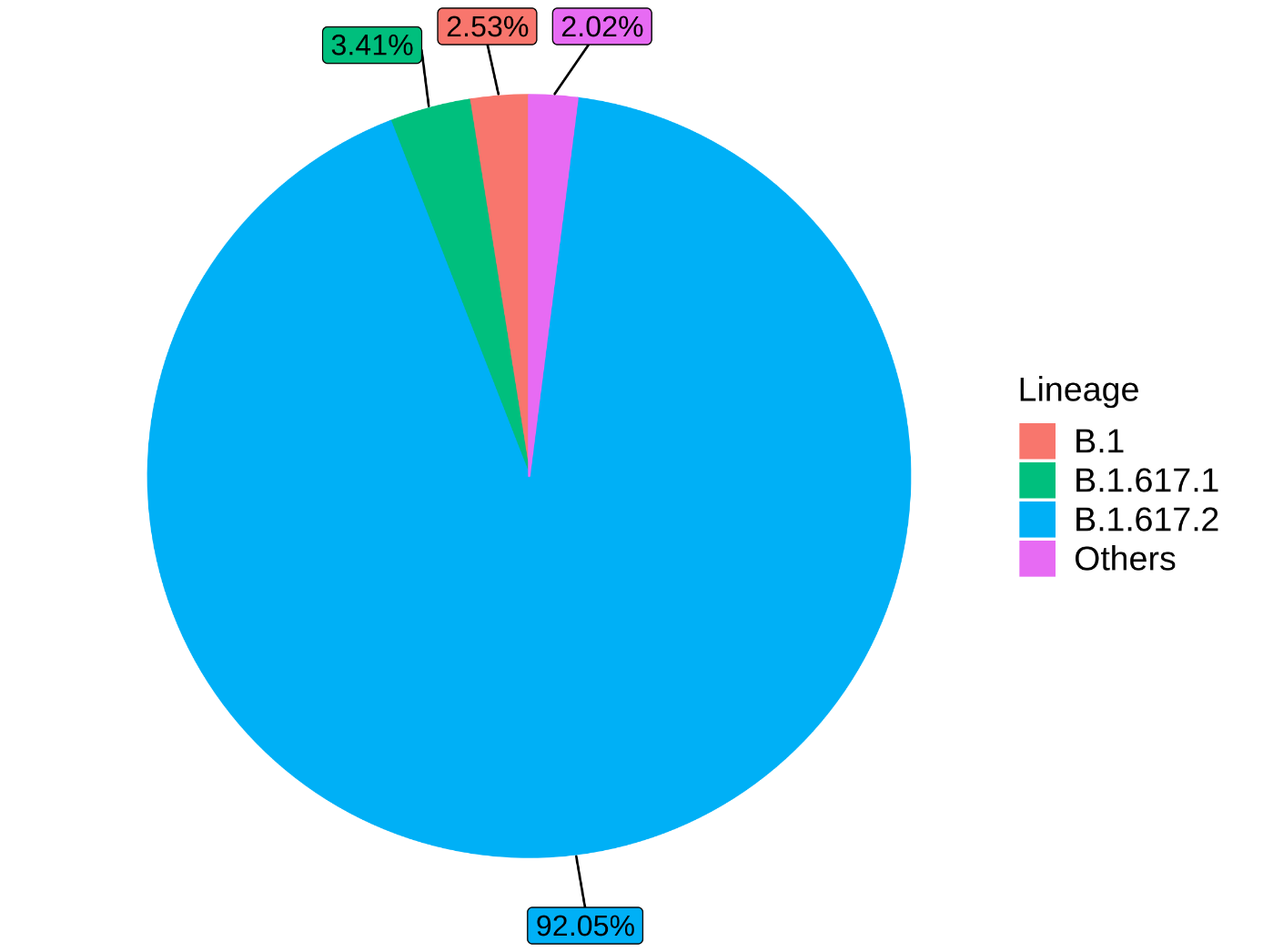


Lineages prevalent in community samples of Telangana, India, in the month of May 2021. Data source: GISAID. Only those genomes which were at least 95% covered are represented (n = 792).


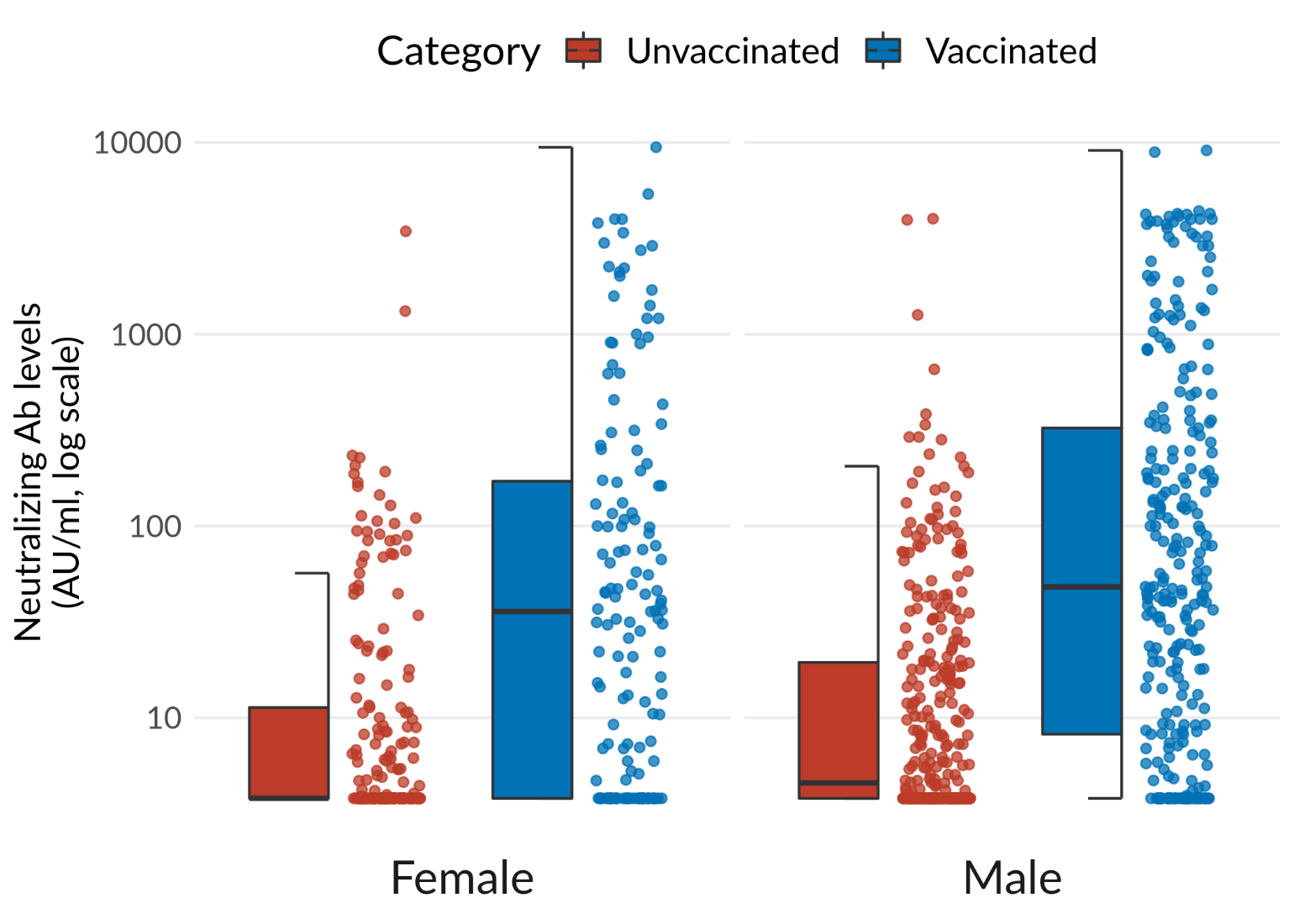


Neutralizing antibody levels in vaccinated and unvaccinated individuals grouped by sex.


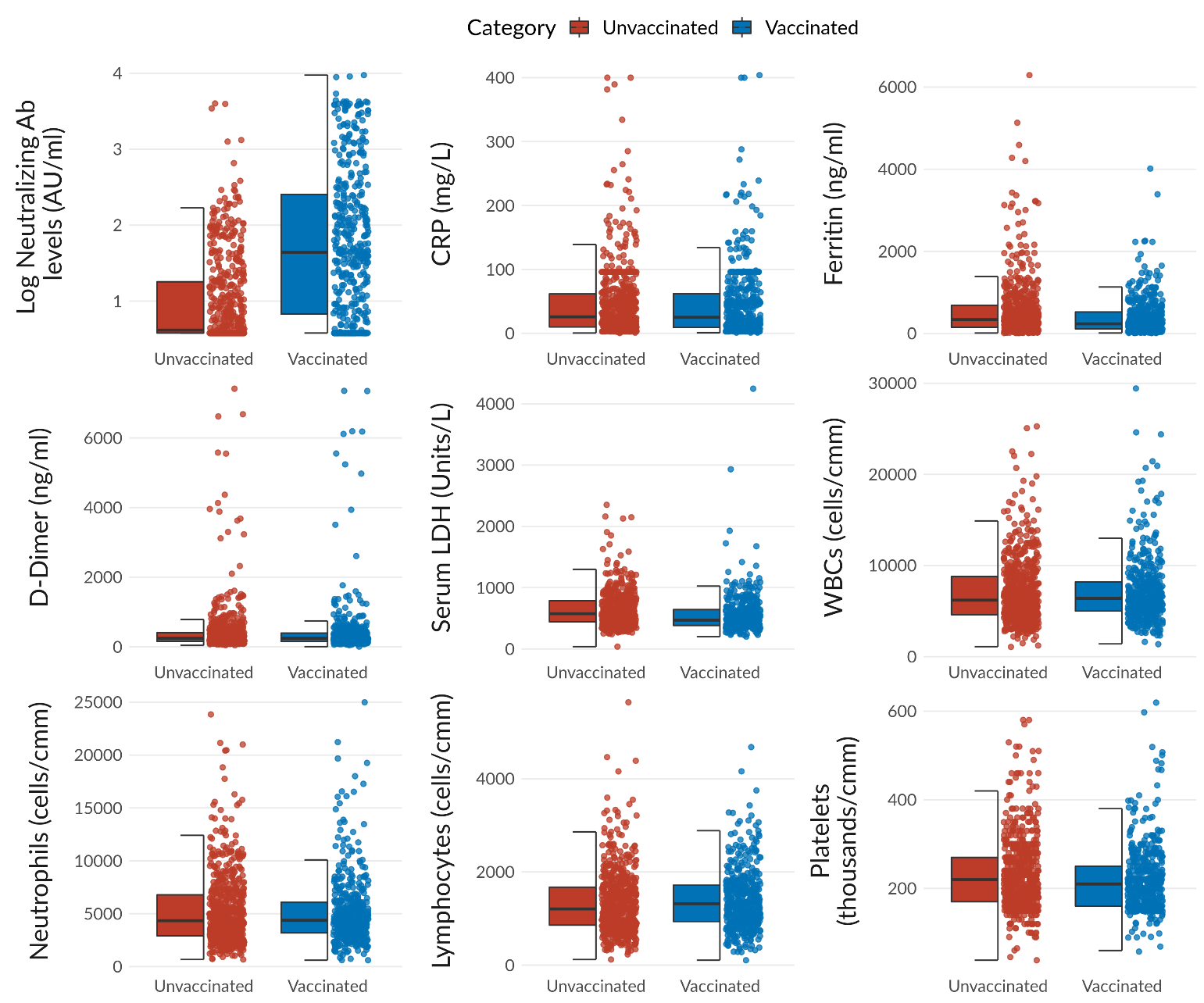


Neutralizing antibody levels and inﬂammatory markers in vaccinated and unvaccinated individuals. The levels of neutralizing antibodies were converted to log10 values before plotting.


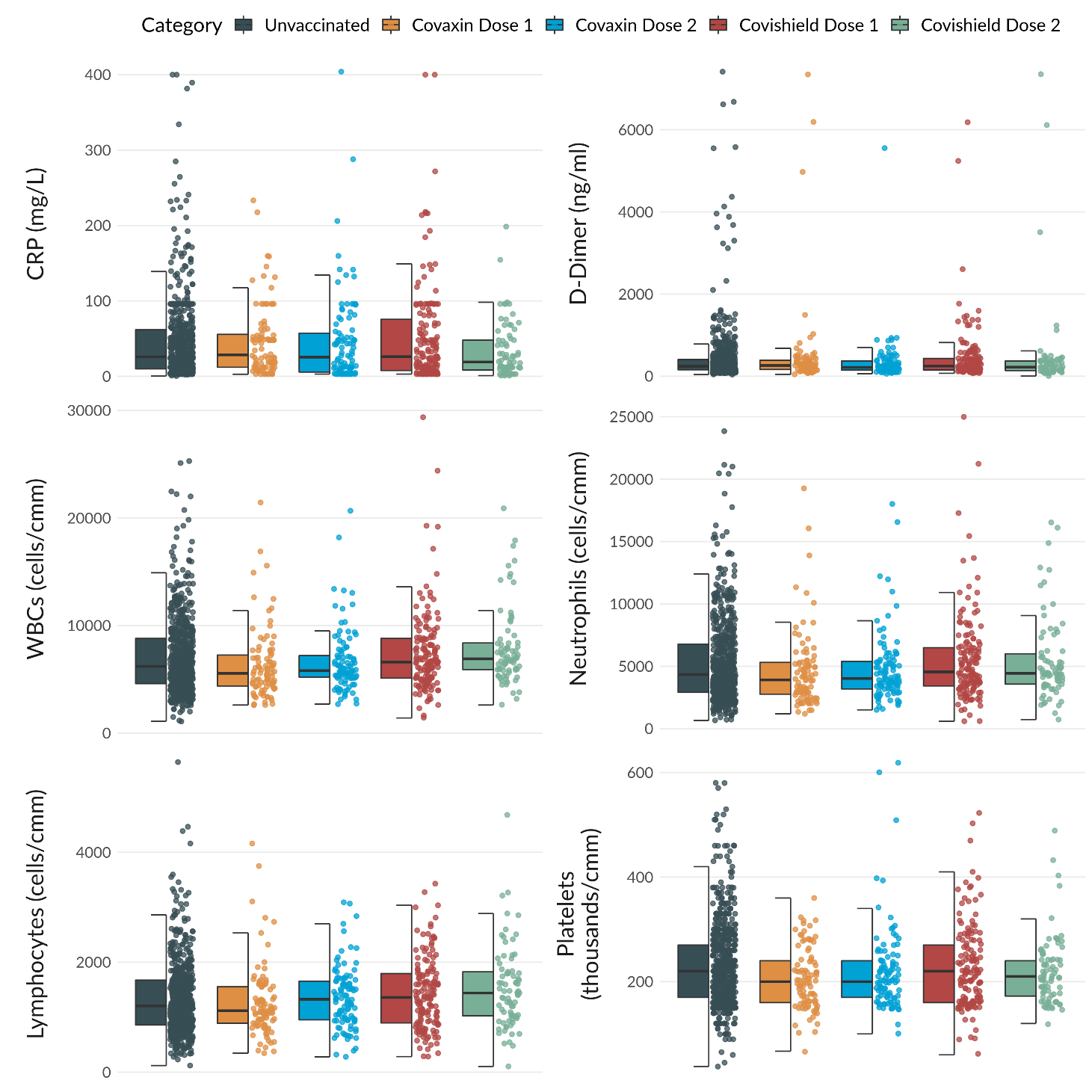


Inﬂammatory markers in vaccinated and unvaccinated individuals, grouped by vaccine type and number of doses.

**Table S1**

Comparison of inﬂammatory markers, comorbidities and outcomes between Covaxin and Covishield (both doses received)

| **Parameter** | **Covaxin (n = 104)** | **Covishield (n=83)** | ***p-value*** |
| --- | --- | --- | --- |
| CRP (mg/L) | 45.51 ± 60.4 (n = 101) | 33.46 ± 37.12 (n = 75) | 0.1046 |
| Ferritin (ng/ml) | 331.91 ± 385.7 (n = 99) | 223.11 ± 187.13 (n = 75) | 0.01539 |
| D-Dimer (ng/ml) | 334.12 ± 561.12 (n = 101) | 481.38 ± 1129.33 (n = 75) | 0.3017 |
| LDH (U/L) | 521.73 ± 206.9 (n = 90) | 516.14 ± 506.71 (n = 64) | 0.9337 |
| Total leukocytes (cells/cu mm) | 6624.75 ± 2909.69 (n = 101) | 7896.15 ± 3541.06 (n = 78) | 0.01114 |
| Neutrophils (cells/cu mm) | 4765.17 ± 2738.71 (n = 101) | 5456.62 ± 3250.23 (n = 78) | 0.1332 |
| Lymphocytes (cells/cu mm) | 1356.85 ± 564.33 (n = 101) | 1532.16 ± 741.88 (n = 77) | 0.08637 |
| Platelets (lakhs/cu mm) | 2.22 ± 0.82 (n = 101) | 2.2 ± 0.65 (n = 78) | 0.8672 |
| Neutralizing antibodies (AU/ml) | 192 ± 607 (n = 92) | 1141 ± 1911 (n = 69) | 1.64e-04 |
| Diabetes/Hypertension | 58 (55.8%)  95% CI - 45-65 | 47 (56.6%)  95% CI - 45-67 | 1 |
| Cardiovascular disease | 7 (6.7%)  95% CI - 2-13 | 7 (8.4%)  95% CI - 3-16 | 0.7817 |
| Respiratory diseases | 2 (1.9%)  95% CI - 0.2-6 | 3 (3.6%)  95% CI - 0.7-10 | 0.6568 |
| Malignancy | 0 (0.0%)  95% CI - 0-3 | 1 (1.2%)  95% CI - 0.03-6 | 0.4439 |
| Kidney disease | 2 (1.9%)  95% CI - 0.2-6 | 4 (4.8%)  95% CI - 1-11 | 0.4088 |
| Chronic liver disease | 0 (0.0%)  95% CI - 0-3 | 1 (1.2%)  95% CI - 0.03-6 | 0.4439 |
| ICU need at admission | 3 (2.9%)  95% CI - 0.6-8 | 0 (0.0%)  95% CI - 0-3 | 0.2555 |
| Severity | 3 (2.9%)  95% CI - 0.6-8 | 1 (1.2%)  95% CI - 0.03-6 | 0.6305 |
| Severe disease/ ICU requirement at  admission | 6 (5.8%)  95% CI - 2-12 | 1 (1.2%)  95% CI - 0.03-6 | 0.1346 |
| ICU requirement during the  hospitalization | 6 (5.8%)  95% CI - 2-12 | 3 (3.6%)  95% CI – 0.7-10 | 0.7333 |
| Ventilatory support | 6 (5.8%)  95% CI - 2-12 | 2 (2.4%)  95% CI - 0.2-9 | 0.3037 |
| Thrombotic complications | 3 (2.9%)  95% CI - 0.6-8 | 3 (3.6%)  95% CI - 0.7-10 | 1 |
| Acute kidney injury | 4 (3.8%)  95% CI - 1-9 | 4 (4.8%)  95% CI - 1-11 | 1 |
| RRT | 2 (1.9%)  95% CI - 0.2-6 | 1 (1.2%)  95% CI - 0.03-6 | 1 |
| Death | 5 (4.8%)  95% CI - 1-10 | 1 (1.2%)  95% CI - 0.03-6 | 0.2291 |
